# Using an Electronic Tablet to Assess Patients’ Home Environment by Videoconferencing Prior to Hospital Discharge: Protocol for a Mixed-Methods Feasibility and Comparative Study

**DOI:** 10.2196/11674

**Published:** 2019-01-14

**Authors:** Karine Latulippe, Véronique Provencher, Katia Boivin, Claude Vincent, Manon Guay, Dahlia Kairy, Ernesto Morales, Marc-André Pellerin, Dominique Giroux

**Affiliations:** 1 Center of Excellence on Aging Quebec Quebec, QC Canada; 2 Department of Teaching and Learning Studies Université Laval Quebec, QC Canada; 3 School of Rehabilitation Université de Sherbrooke Sherbrooke, QC Canada; 4 Center for Research on Aging Sherbrooke, QC Canada; 5 CHU de Québec-Université Laval Quebec, QC Canada; 6 Department of Rehabilitation Université Laval Quebec, QC Canada; 7 Center for Interdisciplinary Research in Rehabilitation and Social Integration Quebec, QC Canada; 8 School of Rehabilitation Université de Montréal Montréal, QC Canada; 9 Center for Interdisciplinary Research in Rehabilitation of Metropolitan Montreal Montréal, QC Canada

**Keywords:** caregivers, feasibility, mixed-methods, mobile videoconferencing, mobile phone, occupational therapists

## Abstract

**Background:**

Occupational therapists working in hospitals are usually involved in discharge planning to assess patients’ safety and autonomy upon returning home. However, their assessment is usually done at the hospital due to organizational and financial constraints. The lack of visual data about the patients’ home may thus reduce the appropriateness and applicability of the support recommended upon discharge. Although various technological tools such as mobile devices (mobile health) are promising methods for home-based distance assessment, their application in hospital settings may raise several feasibility issues. To our knowledge, their usefulness and added value compared to standard procedure have not been addressed yet in previous studies. Moreover, several feasibility issues need to be explored.

**Objective:**

This paper aims to (1) document the clinical feasibility of using an electronic tablet to assess the patient's home environment by mobile videoconferencing and (2) explore the added value of using mobile videoconferencing, compared to the standard procedure.

**Methods:**

A feasibility and comparative study using a mixed-methods (convergent) design is currently undergoing. Six occupational therapists will assess the home environment of their patients in the hospital setting: they will first perform a semistructured interview (a) and then use mobile videoconferencing (b) to compare “a versus a+b.” Interviews with occupational therapists and patients and their caregivers will further explore the advantages and disadvantages of mobile videoconferencing. Two valid tools are used (the Canadian Measure of Occupational Performance and the telehealth responsivity questionnaire). Direct and indirect time is also collected.

**Results:**

The project was funded in the spring of 2016 and authorized by the ethics committee in February 2017. Enrollment started in April 2017. Five triads (n=4 occupational therapists, n=5 clients, n=5 caregivers) have been recruited until now. The experiment is expected to be completed by April 2019 and analysis of the results by June 2019.

**Conclusions:**

Mobile videoconferencing may be a familiar and easy solution for visualizing environmental barriers in the home by caregivers and clinicians, thus providing a promising and inexpensive option to promote a safe return home upon hospital discharge, but clinical feasibility and obstacles to the use of mobile videoconferencing must be understood.

**International Registered Report Identifier (IRRID):**

DERR1-10.2196/11674

## Introduction

Occupational therapists working in hospitals are usually involved in discharge planning by making recommendations—such as environmental adaptations, assistive technologies, home care services—in order to promote a safe return home. To ensure the applicability and appropriateness of these recommendations, they must consider not only the functional capabilities of their patients, but also the environmental conditions in which they will evolve once they return home. For example, it is often essential for occupational therapists to know the number of stairs to access the home, the kitchen layout and hygiene, the availability of support surfaces in their bathroom, the distance to be covered to get to the toilet, and the thresholds around the house. These visual data are thus crucial to select the type of assistive technologies needed in the home, to provide relevant home care services upon discharge, and even to recommend moving into a new living environment [1,2].

Studies have shown that predischarge home visits help to improve patients’ performance in activities of daily living and quality of life [3] and reduce the risk of falls [4], especially in those with orthopedic problems. However, home visits are not always done prior to discharge. Assessments are therefore generally performed in the hospital, mainly because of organizational and financial constraints. More precisely, the costs and time associated with occupational therapists’ travel to patients’ living environment, as well as the short length of hospitalization, make home assessments difficult to perform before discharge [3].

The use of technologies—such as mobile or portable videoconferencing—is thus an innovative solution, as well as an alternative to predischarge home visit. Specifically, mobile videoconferencing would imply that a patient’s relative moves around the home with an electronic tablet or smartphone using Skype software, in order to allow occupational therapist to assess the home environment (furniture, moving areas, etc) from the hospital setting. Although the use of videoconferencing is recognized to provide distance services such as telemedecine between the hospital and the home [5-9], our recent rapid review (submitted manuscript) only identified 6 studies involving the use of videoconferencing to assess the home environment [10-16]. The work of Sandford et al [14-16] highlighted the use of videoconferencing (including several sophisticated cameras) to adequately identify most (86.4%) of the problems observed during a “standard” home visit (eg, going up or down stairs, using kitchen or bathroom facilities). Moreover, the degree of agreement between the recommendations made by the clinicians to overcome the problems identified was high (*P*<.001) for both types of methods (videoconference vs home visit). Although these studies suggest that the use of videoconferencing is an interesting alternative to conducting a home visit, they have been performed in a nonhospital setting, using technicians to operate complex technological equipment. Their results therefore offer very little information about the feasibility of using a mobile videoconferencing (such as Skype) in a real hospital care context to support discharge planning.

Some disadvantages related to the use of telehealth in “real” contexts of care have been reported: low receptivity of the clinician to accept a change of practice [17], difficulty of some users to understand its functioning [9], cost related to the purchase of equipment [17]. However, these disadvantages are not necessarily applicable to the use of “mobile” videoconferencing. Indeed, it is anticipated that the use of mobile videoconferencing is familiar and simple to use for several relatives at the time of discharge, which suggest a promising and inexpensive solution (Skype is free) to support patients with physical disabilities upon returning home.

Given the need to improve knowledge about the patient's home environment prior to the hospital discharge and the limited evidence currently available for the use of mobile videoconferencing, it is essential to document the feasibility to use mobile videoconferencing in this context of care and to explore its effects using a patient-centered approach. This study has 2 objectives: (1) to document the clinical feasibility of using an electronic tablet to assess the patient's home environment by videoconference and (2) to explore the added value of using mobile videoconferencing, compared to the standard procedure.

## Methods

### Design

**Figure 1:**
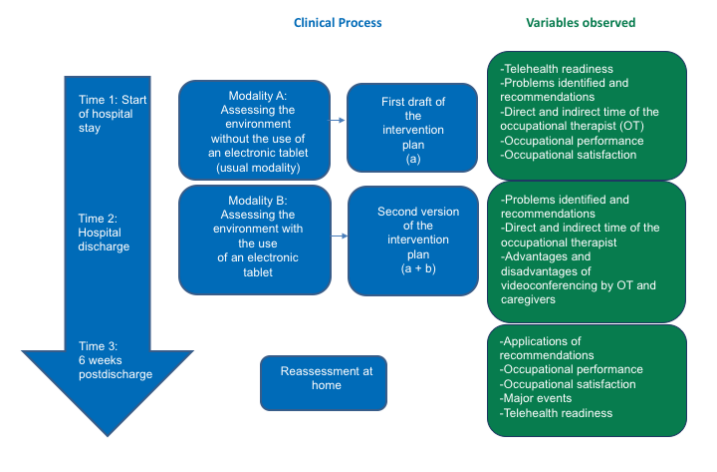
Mapping of the research design. The 3 measurement times are shown in the downward pointing arrow, theclinical process is shown in the middle, and the study variables are shown in green.

A feasibility and comparative study using a mixed-methods (convergent) design is currently undergoing. Qualitative and quantitative data are collected at the same time. Then, these data are merge in the analysis and interpretation of the results [18].The occupational therapist assesses the patient’s home environment from an acute care setting prior to being discharged (*a*) first by conducting an interview (*standard procedure*) and (*b*) then by using mobile videoconferencing, with the aim to compare *a* versus *a*+*b*. Figure 1 shows, from left to right, the 3 measurement times (with a downward pointing arrow), the clinical process (in the middle), and the study variables (in green). These should be assessed with occupational therapists, their patients, and their relatives.

### Technology

Following a positive experience with another clinical project conducted in the same institution, an electronic tablet and Skype software for businesses was selected for this study.

### Sampling

The target sample is 44 participants, including 8 occupational therapists, and 18 patient-relative dyads. As a feasibility study involving an exploratory approach, this number of participants was deemed appropriate [19]. Recruitment takes place in 2 hospitals located in Quebec, Canada among adult patients presenting a loss of functional autonomy mainly due to physical disabilities (orthopedic, neurology). Eligibility criteria are verified for potential participants (patients or relatives) who have agreed to be contacted by a member of the research team.

The inclusion criteria are as follows: (1) for the patient to be hospitalized at the time of recruitment, to have a relative available, and to plan a return home (or residence for seniors); (2) for the family relative to be able to express oneself (in French or English) and move without technical assistance; (3) for the occupational therapist to have at least 1 year of clinical experience. Exclusion criteria are (1) for the patient to have regular follow-up at home by a community-based occupational therapist before the hospitalization and be unable to express themselves and (2) for the relative to be presenting sensory impairments (hearing) likely to interfere with the comprehension of verbal instructions (clinical judgment). Efforts are made to obtain different profiles of participants (age, gender, diagnosis). All participants must be able to consent to the research.

### Ethics Approval

The ethical aspects of this study were approved by the Research Ethics Committee of the Centre intégré universitaire de santé et de services sociaux of Estrie—CHUS (#MP-31-2017-1485) and the Research Ethics Committee of the Quebec University Hospital—Université Laval (#2017-3047).

### Participant Information and Informed Consent

With the authorization of the coordinators of services, the research project is presented to the occupational therapists of the different units targeted by the project by one of the researchers. Occupational therapists interested in participating must sign a consent form previously approved by the 2 ethics committees. Afterwards, when they meet a patient who meets the inclusion criteria and the assessment of the home is relevant, they briefly present the project to the patient and their caregiver and request authorization for a research assistant to contact them. For patients and caregivers who have accepted, the research assistant makes contact with them by phone or in person and presents the research project in detail, insisting that a refusal does not prevent them from having a quality occupational therapy service. For each patient and caregiver who has agreed to participate in the project, a consent form is signed.

### Quantitative Outcomes

Four tools were used to document the quantitative variables:

1. The Evaluation of the Receptivity to Telehealth [20] allows for the evaluation of occupational therapists’ receptivity to the utilization of mobile videoconferencing. This is a French version of a questionnaire validated by clinicians, aiming to assess their receptivity to the introduction of telehealth services. The variables evaluated are the receptivity of the clinician (15/85), the clinician’s commitment or involvement or engagement (35/85), and the receptivity of infrastructures from the clinician’s perspective (35/85). Interpretation of the instrument is as follows: >80 indicates that the practitioners are well positioned to use telehealth; 60-80 indicates that certain factors or items may negatively affect the use of telehealth; and <60 indicates that there are obstacles to the successful use of telehealth by practitioners.
2. A Grid documenting the process of home environment assessment, completed by the occupational therapist, serves to note the time devoted to the evaluation of the environment at the time of hospital discharge (the number of minutes for discussions, arranging an appointment with the relative, and explanations prior to the evaluation) a) with and b) without the use of mobile videoconferencing (see Multimedia Appendix 1) [18].
3. The *Canadian Measure of Occupational Performance* (COPM) [21,22] allows for the measurement of performance and client satisfaction at Times 2 and 3. The COPM is a recognized standardized questionnaire comprising of a semistructured interview format and offering an excellent reliability test-retest [22] with the targeted clientele. A research version is used [23]. This version can be administered over a short period of time (10 minutes), with precise questions for the evaluator and some elements in red that must be filled in by the participant. This will allow for a determination of whether performance and satisfaction have improved between the 2 evaluation periods. A 2-point change is considered a clinically relevant improvement or deterioration [24].
4. Finally, the *Social Readjustment Rating Questionnaire* [25] is used to identify major events (confounding variables) occurring between the 2 points in time or measurement periods. The instrument lists 43 possible events, such as another hospitalization or the reception of home support services different from those recommended during the hospital stay.

### Qualitative Outcomes

Three instruments are used to document the qualitative variables:

1. Grid documenting the characteristics of the intervention plan, completed by the occupational therapist, allows for documentation of the situation, with the help of items to be checked off and short answer questions, problems identified in the client’s home (eg, architectural obstacles), and the resulting recommendations (technical aids or adaptation, the teaching of energy-saving techniques or prevention of falls, reference to home or community support services, etc) a) with and b) without the use of mobile videoconferencing (see Multimedia Appendix 2).
2. A Grid to follow up on recommendations serves to document their applicability immediately after discharge from the hospital (completed by the occupational therapist), as well as 6 weeks later (completed by a research assistant; see Multimedia Appendix 3). For example, following the occupational therapist’s recommendation to use a 19-inch wheeled commode chair over the toilet, it is specified that the patient procured this equipment and continues to use it. The grid was pretested with an occupational therapist not involved in the project before the study begins.
3. A *semistructured individual interview* was conducted to document the perceptions of the occupational therapist and the relative concerning the advantages and disadvantages related to the utilization of mobile videoconferencing. The semistructured interview follows the rules of qualitative research [26], and the interview guide was pretested [27]. The thematic variables documented are previous and current experience with the use of mobile videoconferencing, the obstacles and problems encountered with the use of mobile videoconferencing during the study, and the perceived advantages of using this method. The interview will be audio recorded.

The clinical and sociodemographic profile of patients (gender, age, diagnosis, comorbidities, admission date, and discharge date), caregivers (gender, age, relationship to the patient, and familiarity with the technology: weak, moderate, or substantial) and occupational therapists (gender, years of experience in the profession, and number of years in the program or department) is also collected.

### The Sequence of Research Stages

- Time 1 (Ti, interview): The researcher meets the previously recruited patient-relative dyad to confirm their participation and sign the consent form. Each occupational therapist involved in the project completes the *Telehealth Readiness Assessment* prior to the beginning of the study and then convene with the relative of a moment to perform videoconferencing prior to discharge. The occupational therapist then collects data on the clinical and sociodemographic profile of the participants and conducts the semistructured interview (as per the standard procedure) to assess the client's home environment. The occupational therapist gathers information about the problems identified, the recommendations made, and the time required (direct-indirect) to carry out the interview (*Intervention plan*). The research assistant administers the COPM to the patient prior to discharge.
- Time 2 (T2, mobile videoconferencing): The occupational therapist uses mobile videoconferencing to remotely assess the patients’ home environment with the help of the relative. The latter is asked to move around with the device (electronic tablet or smartphone) in the various rooms of the home according to occupational therapist’s indications. The relative was previously trained by the occupational therapist to become more familiar with the use of mobile videoconferencing. Using the notes recorded at the time of the previous assessment (interview), the occupational therapist must complete the home assessment with mobile videoconferencing within 48 hours in order to review the problems identified and modify the recommendations made upon discharge. Recommendations based on this last revised intervention plan are those transmitted to the patient. The problems identified, the recommendations made, and the time (direct-indirect) required by the occupational therapist to carry out the mobile videoconferencing are documented. Finally, the researcher assistant conducts an interview with the occupational therapist and a second with the family relative to document their perception of the mobile videoconferencing (Individual Interview Guide).
- Time 3 (T3, 6 weeks postdischarge): The research assistant goes to the home to document (1) the perception of the applicability of the recommendations and whether they are applied or currently used (15-minute follow-up chart and 15-minute interview with the patient) and (2) performance and satisfaction (COPM) and major events since hospital discharge (Social Readjustment Rating Questionnaire). Upon completion of the study, the occupational therapist completes again the Telehealth Readiness Assessment.

### Data Analysis

Descriptive analyses are performed to report the clinical and sociodemographic profile, the direct or indirect time, the nature or number of unforeseen events, the receptivity, and the occupational performance and satisfaction scores (means, frequencies, SDs). Wilcoxon tests are used to compare the receptivity scores collected from occupational therapists at the beginning (T1, before the first client) and at the end of the study (T3, after the last client). This will allow us to explore the hypothesis that an improvement in receptivity supports the feasibility of the project. The same test is used to compare COPM scores (T1 vs T3) to explore the hypothesis of postdischarge changes in patient performance and satisfaction.

A qualitative analysis using analytical questioning [28] is currently being carried out based on the verbatim of interviews. The purpose is to identify the variations in themes related to the subquestions emerging from 3 sources: the interview guide, the intervention plan (recommendations made at discharge), and the follow-up grid (recommendations applied postdischarge). N'Vivo software is used to support this analysis. The analysis highlights how respondents' perceptions differ from one to another and how the problems identified and the recommendations made based on the interview (*a*) are different from those made with the combination with mobile videoconferencing (*a*+*b*). This helps to document the link between the recommendations made, the application of the recommendations, and the improvement of the occupational performance or satisfaction, taking into account unforeseen events. Crossing qualitative and quantitative data from a matrix created by the N'Vivo software will be performed to improve the understanding and interpretation of the data [29].

### Participant Confidentiality and Security

All data concerning the participants (occupational therapist, patient, and caregiver) is considered confidential. The documents are coded by patient by the research assistants who meet the triad. The recordings of the interviews are transcribed by a research assistant and denominated. Apart from the research assistants conducting the data collection, individuals will not be recognized through the results. The raw data (grids, questionnaires, records) are stored on a secure server at the Integrated University Health and Social Services Center of the Capitale-Nationale and in a locked file in a secure research center. The data are kept for 5 years. Mobile videoconference is not recorded. Apart from the occupational therapist who performed the home assessment, no one can see the patient's home. The Skype software for businesses was chosen because it complies with the safety level regulations of the Quebec Health and Social Services Network. In addition, the devices used (electronic tablet) are only used for this project. Some occupational therapists use their office computer at the hospital where they work to conduct the home visit. These computers are also secure.

## Results

The project was funded in the spring of 2016 and authorized by the ethics committee in February 2017. Enrollment started in April 2017. Five triads (4 occupational therapists, 5 clients, and 5 caregivers) have been recruited until now. The experiment is expected to be completed by April 2019 and analysis of the results by June 2019.

## Discussion

The current protocol describes a mixed-methods feasibility and comparative study to document the clinical feasibility of using an electronic tablet to assess the patient's home environment by mobile videoconferencing and to explore the added value of using mobile videoconferencing compared to the standard procedure (interview). This study uses 4 ways to document the quantitative variables and 3 ways to document the qualitative variables. Crossing qualitative and quantitative data from a matrix will be performed to improve the understanding and interpretation of the data.

Mobile videoconferencing may be a familiar and easy solution for visualizing environmental barriers in the home by caregivers and clinicians, thus providing a promising and inexpensive option to promote a safe return home upon hospital discharge, but clinical feasibility and obstacles to the use of mobile videoconferencing must be understood.

## Appendix

Multimedia Appendix 1 Grid documenting the process of home environment assessment.

Multimedia Appendix 2 Grid documenting the Characteristics of the Intervention Plan.

Multimedia Appendix 3 Grid to follow up on recommendations.

## Abbreviations

COPM: Canadian Measure of Occupational Performance
