# Supplementary material for: Using an Electronic Tablet to Assess Patients’ Home Environment by Videoconferencing Prior to Hospital Discharge: Protocol for a Mixed-Methods Feasibility and Comparative Study
Source: JMIR Res Protoc. 2019 Jan 14;8(1):e11674. doi: 10.2196/11674 (PMC6682277; doi:10.2196/11674)
Supplement: Multimedia Appendix 1 [file resprot_v8i1e11674_app1.pdf]

**Multimedia Appendix 1: Grid documenting the process of home environment assessment**

**Participant: ____________________________________ OT: ____________________________________**

Enter here all the steps taken for the assessment of your patient's home with the tablet. You must specify whether a planning process OR an evaluation process using the keywords below.

| | Date | Reason (choose one or the other) | | Duration | Several tests needed to complete this process? |
|---|---|---|---|---|---|
| 1 | | ☐Planning process<br>☐ making an appointment<br>☐in person<br>☐by phone<br>☐Education related to the tablet<br>☐Equipment loan<br>☐Other : | ☐Evaluation process<br>☐Formulate instructions<br>☐Viewing the environment<br>☐Questions<br>☐Issue(s) encountered: (specify):<br>☐Other: | | ☐Yes<br>☐No, specify : |
| 2 | | ☐Planning process<br>☐ making an appointment<br>☐in person<br>☐by phone<br>☐Education related to the tablet<br>☐Equipment loan<br>☐Other : | ☐Evaluation process<br>☐Formulate instructions<br>☐Viewing the environment<br>☐Questions<br>☐Issue(s) encountered: (specify):<br>☐Other: | | Yes<br>☐No, specify : |
