# Supplementary material for: Using an Electronic Tablet to Assess Patients’ Home Environment by Videoconferencing Prior to Hospital Discharge: Protocol for a Mixed-Methods Feasibility and Comparative Study
Source: JMIR Res Protoc. 2019 Jan 14;8(1):e11674. doi: 10.2196/11674 (PMC6682277; doi:10.2196/11674)
Supplement: Multimedia Appendix 2 [file resprot_v8i1e11674_app2.pdf]

Multimedia Appendix 2: Grid documenting the Characteristics of the Intervention Plan

**Problems identified during functional assessment in occupational therapy before the use of mobile videoconferencing**	**Recommendations before the use of mobile videoconferencing Date:**	**Applicability: Is the initial recommendation is applicable to the use of mobile videoconferencing?**	**Recommendations after the use of mobile videoconferencing Date:**	**Explain the change, if it's the case and why.**
Example: 1. Difficulty in transfers to the toilet	*4 " toilet seat + safety support frame*	☐**Yes**<br>☒**No**	*High chair 19" on wheels placed above the toilet*	*The space between the toilet and the bath is too small and the safety support frame would block access to the bathing board during the transfer. The toilet commode on wheels will be more easily moved by the spouse to facilitate access to the bath.*
		☐**Yes**<br>☐**No**		
		☐**Yes**<br>☐**No**		
