# Supplementary material for: Using an Electronic Tablet to Assess Patients’ Home Environment by Videoconferencing Prior to Hospital Discharge: Protocol for a Mixed-Methods Feasibility and Comparative Study
Source: JMIR Res Protoc. 2019 Jan 14;8(1):e11674. doi: 10.2196/11674 (PMC6682277; doi:10.2196/11674)
Supplement: Multimedia Appendix 3 [file resprot_v8i1e11674_app3.pdf]

Multimedia Appendix 3: Grid to follow up on recommendations

Participant name: ____

OT name: ____

Date: ____

**Recommendation following the use of mobile videoconferencing**	**Application during follow-up 6 weeks? Specify.**
1. *Example: High chair 19" on wheels placed above the toilet*	*1. Yes, Mr. says to use it with each use of the toilet.*
**2.**	**2.**
**3.**	**3.**
